# Intramuscular Hemangioma of Thyrohyoid Muscle

**DOI:** 10.1155/2016/7158691

**Published:** 2016-08-28

**Authors:** Ramesh Parajuli, Suman Thapa, Sushna Maharjan

**Affiliations:** ^1^Department of Otorhinolaryngology, Chitwan Medical College Teaching Hospital, P.O. Box 42, Bharatpur 10, Chitwan, Nepal; ^2^Department of Pathology, Chitwan Medical College Teaching Hospital, P.O. Box 42, Bharatpur 10, Chitwan, Nepal

## Abstract

Intramuscular hemangiomas are rare benign vascular neoplasms. IMH accounts for less than 1% of all hemangiomas. These neoplasms commonly occur in trunk and extremities but are rare in head and neck region. The present case is a 17-year-old female patient, who presented with a painless, slowly enlarging mass in left sided upper neck for 4 years. Investigations were suggestive of vascular neoplasm. She underwent excision of the mass in toto under general anesthesia. Postoperative period was uneventful. Histopathological examination of the mass revealed it as mixed type of intramuscular hemangioma. She did not have any signs of recurrences on her last follow-up at 6 months postoperatively. This case report discusses the rare IMH arising from thyrohyoid strap muscle.

## 1. Introduction

Hemangiomas are benign vascular neoplasms which usually occur in the skin and mucosa. However, intramuscular hemangioma (IMH) is a rare neoplasm accounting for less than one percent of all hemangiomas [1]. IMH are frequently found in trunk and extremities but are uncommon in the head and neck region. The masseter and the trapezius muscles are the most commonly involved muscles in this region with thyrohyoid muscle being the uncommon site. Thyrohyoid is an infrahyoid type of strap muscle which is a striated muscle in the neck. Its action is to depress the hyoid bone thus elevating the larynx. There are only a few cases of intramuscular haemangioma arising from this muscle, reported in the literature [2]. We report a case of intramuscular haemangioma of thyrohyoid muscle.

## 2. Case Report

**Figure 1:**
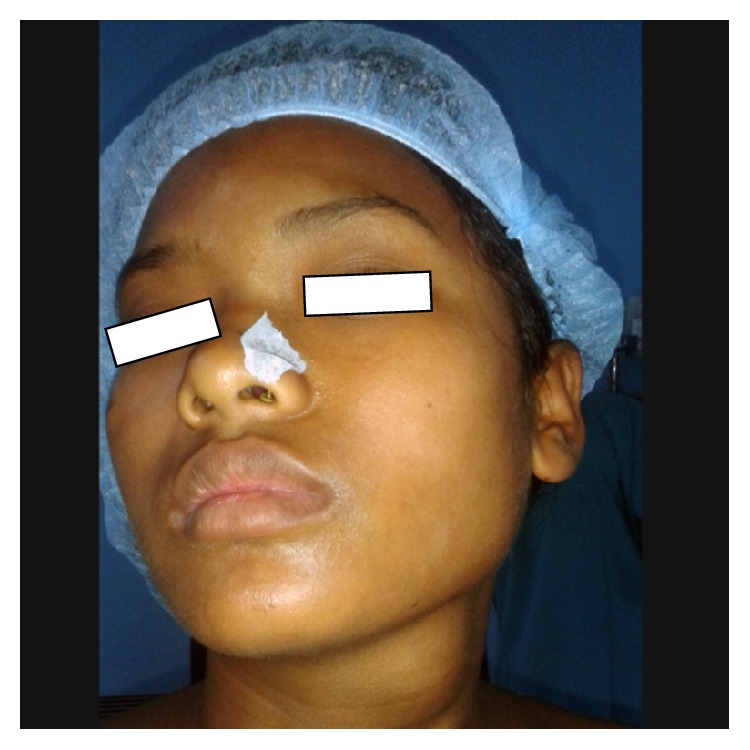
Preoperative photograph showing mass in the left sided upper neck.

A 17-year-old female presented with a left sided upper neck swelling which was insidious in onset and was painless and progressively increasing in size over the past 4 years (Figure 1). It was not associated with dysphagia, dyspnea, or dysphonia. There was no history of trauma. It was not associated with similar swellings in other body parts. Her head and neck examination revealed a 4 × 3 cm swelling in left anterolateral region of upper neck. It was soft, not tender, nonpulsatile, and nonexpansible on Valsalva maneuver. There was no palpable thrill. Oral cavity examination did not reveal any abnormality. The overlying skin was normal in color without local rise of temperature and it was free from the underlying swelling. Her medical and family history was unremarkable. Based on the history and clinical examination, provisional diagnosis of branchial cyst was made.

**Figure 2:**
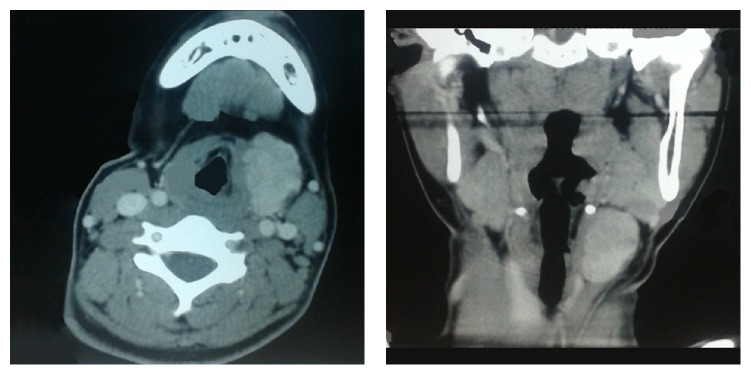
CT scan showing the soft tissue mass in the neck (axial and coronal view).

Ultrasonography revealed a well defined hyperechoic mass, suggested as a highly vascular mass. Fine needle aspiration cytology (FNAC) examination was suggestive of vascular lesion. Computed tomography (CT) scan showed an inhomogeneous hypodense lesion lying in close approximation to left ala of thyroid cartilage (Figure 2). There was no obvious separation of the mass from the strap muscle. The differential diagnoses suggested from the CT scan report were vascular malformation and hemangioma.

**Figure 3:**
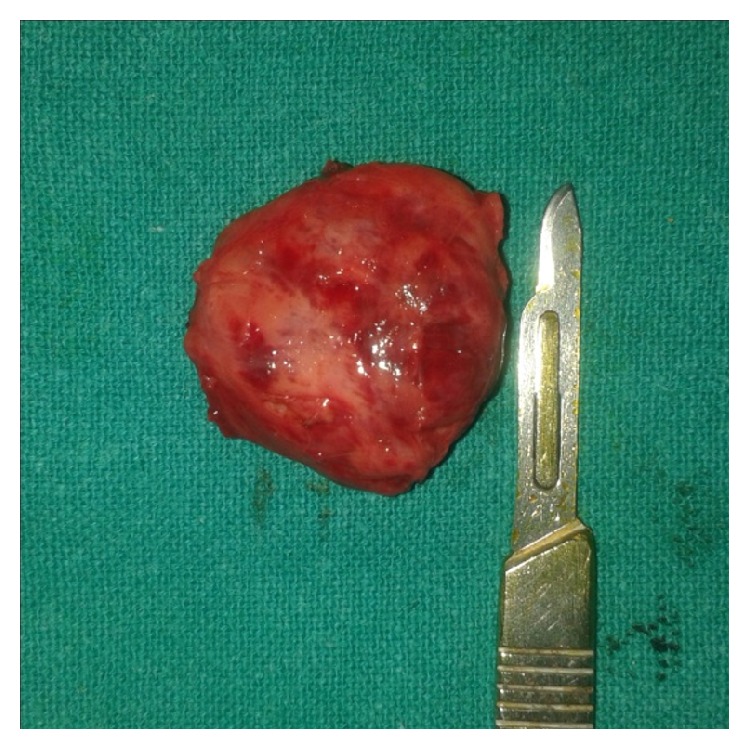
Excised (surgical) specimen.

**Figure 4:**
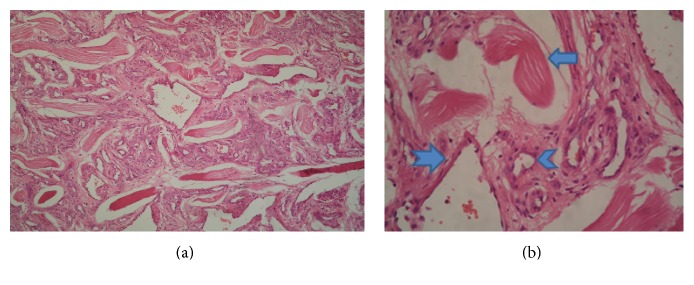
(a) Histopathological examination (10x powers): the field shows numerous capillary-sized vessels and large dilated vessels extending between skeletal muscle fibers. (b) Histopathological examination (40x powers): skeletal muscle fiber (arrow), capillary-sized vessel (arrow head), and dilated large blood vessel (notched arrow).

Under general anesthesia, skin incision was given along the skin crease on top of the swelling. Blunt dissection was then carried out gently. Intraoperatively, the mass was found to be closely adherent to the left lamina of thyroid cartilage. The mass was then mobilized from the surrounding structures by ligating the feeding vessels separately and dissecting the soft tissue attachments. There was minimal blood loss during the procedure and the mass was removed in toto (Figure 3). Hemostasis was secured. Corrugated rubber drain was placed to prevent hematoma formation. Wound was sutured in layers using absorbable and nonabsorbable sutures. Pressure dressing was applied. The specimen was sent for histopathological examination (HPE). On hematoxylin and eosin staining, it showed numerous capillary-sized vessels and large dilated vessels extending between skeletal muscle fibers; thus, it was diagnosed as mixed type of intramuscular haemangioma (Figure 4). There was no change in her voice postoperatively. The postoperative period was uneventful. She was discharged on the 6th postoperative day following removal of the suture. There was no sign of recurrence on follow-up at 6 months postoperatively.

## 3. Discussion 

Haemangiomas are mostly tumors of infancy, more frequently arising from mucosal and cutaneous surfaces [3]. Intramuscular haemangiomas (IMH) are uncommon vascular neoplasms in the head and neck region. These hemangiomas are nonencapsulated benign neoplasms containing different size of vascular channels with variable amounts of adipose, fibrous, and myxoid tissue components. The intramuscular hemangiomas account for about 1% of all hemangiomas. Of the IMH, only 14 to 21% are found in the head and neck region [2]. Exact etiology of hemangioma is not known. There are various theories suggested by various authors regarding its origin such as congenital, traumatic, and hormonal theories. IMH have been divided into 3 different subtypes; these are capillary, cavernous, and mixed types. Among these types, the capillary type is the most common, usually having a short clinical history of small painless swelling. Cavernous type of IMH usually has a longer history with the lesion being slightly larger in size and the vessels being larger in diameter. In case of mixed variant of IMH, there is a combination of capillary and large dilated vessels. Histologically, the mixed type is similar to cavernous type and its clinical features also resemble to it. Among the subtypes of IMH, mixed type has the highest recurrence rate [2].

Diagnosis of IMH is not easy because it is an uncommon disease without any characteristic findings on clinical presentation. These are benign congenital tumors that often remain undetected for a long time. These neoplasms usually present as progressively enlarging mass which may or may not be associated with pain. There are various diagnostic tools that can be used such as ultrasonography, FNAC CT, MRI, and arteriography. Ultrasonography, particularly the color Doppler sonography, often shows it as a well defined hypoechoic mass with heterogenous echotexture. FNAC is often nonconclusive as it yields fresh blood on aspiration. Radio imaging investigations can be helpful in certain cases where there are dilated and tortuous vascular spaces, thrombus, and phleboliths especially in cavernous IMH. CT scan may reveal enhancing well defined intramuscular mass but often it is unable to define tissue planes. MRI can be useful in soft tissue mass like IMH, where there is usually high intensity signals on T2 weighted MRI which is due to increased fluid content due to increased vascularity in IMH. In our case, the patient did not undergo MRI investigation as the patient was unable to afford it. When the swelling is in the neck, it may be confused as lipoma, lymphangioma, or vascular malformations on ultrasonography.

IMH has been found to occur in masseter, trapezius, sternocleidomastoid, temporalis, and orbital muscles. Cases of IMH arising from the posterior and middle scalenus and digastric muscles have also been reported in the literature [4, 5]. Thus, hemangioma should be considered as a differential diagnosis in case of head and neck soft tissue swellings. It can be ruled out from other lumps by its features such as increased vascularity and high blood flow velocity in that mass. Similarly, it can be distinguished from arteriovenous malformations by the presence of solid parenchymal components [6]. Lipoma should also be considered as a differential diagnosis as it is painless and progressive mass which is often asymptomatic. However, lipoma is rare in the head and neck region. On CT scan, lipoma appears as homogenous and low density mass. As the swelling appears in upper lateral neck close to the anterior border of sternocleidomastoid muscle, it may be confused as branchial cyst. However, the fine needle aspiration cytology along with the ultrasonography can give the diagnosis of branchial cyst without much dilemma. Similarly, lymphangioma can also present as painless, soft tissue swelling in the neck but it is present since birth so it is usually detected by the age of 2 years.

Unlike cutaneous and mucosal hemangiomas, intramuscular hemangiomas usually do not regress spontaneously. Management of IMH depends on its location, extent, growth rate, accessibility, age of the patient, and cosmetic factors [7]. Various treatment methods have been used for the treatment of hemangiomas such as intralesional steroid injection, sclerotherapy using ethanol or sodium tetradecyl sulphate, cryotherapy, vascular ligation, embolization, and excision. Treatment of choice for IMH includes excision of the lesion along with the adequate surrounding normal tissue due to lack of encapsulation and infiltrative nature of the hemangioma into surrounding muscular tissue plane. However, the recurrence rate ranging from 9 to 28% has been reported following surgical excision [2]. There is no malignant transformation of such lesions. However, due to the relatively higher chance of recurrence especially in mixed type of IMH, patients need to be followed up to detect and treat recurrence.

## 4. Conclusion

IMH are rare benign vascular neoplasm of head and neck region which usually present as painless and very slowly progressive swelling. The preoperative diagnosis is often difficult. Radio imaging and color Doppler sonography are useful for diagnosis of such lesions. Surgical resection of the lesion with a small cuff of surrounding muscle is the treatment of choice; that can be done without any excessive bleeding from this highly vascular mass.
